# Optimization of Early Steps in Oncolytic Adenovirus ONCOS-401 Production in T-175 and HYPERFlasks

**DOI:** 10.3390/ijms20030621

**Published:** 2019-01-31

**Authors:** Lukasz Kuryk, Anne-Sophie W Møller, Antti Vuolanto, Sari Pesonen, Mariangela Garofalo, Vincenzo Cerullo, Magnus Jaderberg

**Affiliations:** 1 Targovax Oy, Clinical Science, 00180 Helsinki, Finland; 2 National Institute of Public Health–National Institute of Hygiene, Department of Virology, 00-0791 Warsaw, Poland; 3 Drug Research Program, ImmunoVirothearpy Lab, Faculty of Pharmacy, University of Helsinki, 00014 Helsinki, Finland; mariangela.garofalo@unimi.it (M.G.); vincenzo.cerullo@helsinki.fi (V.C.); 4 Targovax ASA, Clinical Science, 0283 Oslo, Norway; anne-sophie.moller@targovax.com (A.-S.W.M.); magnus.jaderberg@targovax.com (M.J.); 5 Targovax Oy, CMC, 00180 Helsinki, Finland; antti.vuolanto@gmail.com; 6 Targovax Oy, R&D, 00180 Helsinki, Finland; sari.pesonen@valotx.com; 7 Department of Oncology and Hemato-Oncology, Center of Excellence on Neurodegenerative Diseases, University of Milan, 20133 Milan, Italy

**Keywords:** oncolytic adenovirus, CD40L, productivity, benzonase, manufacturing, optimization, cancer, MOI, harvesting time, virus productivity

## Abstract

Oncolytic adenoviruses can trigger lysis of tumor cells, induce an antitumor immune response, bypass classical chemotherapeutic resistance strategies of tumors, and provide opportunities for combination strategies. A major challenge is the development of scalable production methods for viral seed stocks and sufficient quantities of clinical grade viruses. Because of promising clinical signals in a compassionate use program (Advanced Therapy Access Program) which supported further development, we chose the oncolytic adenovirus ONCOS-401 as a testbed for a new approach to scale up. We found that the best viral production conditions in both T-175 flasks and HYPERFlasks included A549 cells grown to 220,000 cells/cm^2^ (80% confluency), with ONCOS-401 infection at 30 multiplicity of infection (MOI), and an incubation period of 66 h. The Lysis A harvesting method with benzonase provided the highest viral yield from both T-175 and HYPERFlasks (10,887 ± 100 and 14,559 ± 802 infectious viral particles/cell, respectively). T-175 flasks and HYPERFlasks produced up to 2.1 × 10^9^ ± 0.2 and 1.75 × 10^9^ ± 0.08 infectious particles of ONCOS-401 per cm^2^ of surface area, respectively. Our findings suggest a suitable stepwise process that can be applied to optimizing the initial production of other oncolytic viruses.

## 1. Introduction

Oncolytic viruses (OVs) selectively replicate and lyse cancer cells; spreading within the tumor mass; circulating into distant metastases; and not significantly harming normal cells. OVs can exhibit natural tumor-selective tropism (reovirus) [1] or be genetically modified for cancer cell-restricted replication (adenovirus; poliovirus; herpes simplex; vaccinia; Newcastle disease virus; measles) [2,3].

Oncolytic adenoviruses constitute a class of biologics that can trigger lysis of tumor cells, release damage-associated molecular patterns (DAMPs), which help induce an antitumor immune response, circumvent classical chemotherapeutic resistance strategies of tumors, and provide opportunities for combination strategies [4,5,6,7,8,9,10,11,12,13,14,15,16,17,18]. Oncolytic adenoviruses often have a mutated or deleted *E1A* gene, which is required for adenoviral replication. E1A proteins are responsible for dissociation of the retinoblastoma (Rb)/E2F proteins, resulting in activation of free transcription factor E2F. Many of the commonly investigated oncolytic adenoviruses have a 24bp deletion in the *E1A* gene (Δ24), which disrupts the retinoblastoma (Rb) binding domain, arresting virus replication [8].

In addition, oncolytic adenoviruses can augment or broaden the immune response by expressing a transgene with inflammatory or immune stimulatory properties, such as granulocyte-macrophage colony-stimulating factor (GM-CSF) [19,20], CD40L [4,21,22,23,24,25], and IL-12 and IL-18 [26]. Adenovirus 5 was the most commonly explored serotype, but its receptor (CAR) is downregulated in many tumor types and it has a strong tropism for the liver which sequesters most of the intravenously (i.v.) administered dose. Many oncolytic adenoviruses incorporate chimeric fiber knobs so they have altered specificity by utilizing receptors for alternate serotypes [8,19,22,23,27]. In comparison to Ad5 vectors, oncolytic Ad with an RGD motif interacts more efficiently with the integrins expressed on ovarian and prostate cancers and gliomas [5]. Oncolytic Ad with a chimeric Ad3 fiber knob interacts more efficiently with desmoglein (DSG), which is overexpressed in many epithelial cancers [28,29].

Like all adenoviruses [30], one of the major challenges of moving targeted adenoviruses through clinical trials is the development of scalable production methods for generating viral seed stocks and sufficient quantities of clinical grade viruses in a suitable formulation [11]. We chose to use the oncolytic adenovirus ONCOS-401 as a model because its intratumoral administration into progressing advanced solid tumors in nine patients in a regulated Advanced Therapy Access Program (ISRCTN10141600) showed disease control in 83% of the evaluable patients [22]. ONCOS-401 is an oncolytic adenovirus with three modifications [23]. First, its *E1A* gene is driven by the human telomerase (hTERT) promoter [23], which is commonly overexpressed in many tumors. Second, ONCOS-401 features an Ad5/3 chimeric fiber knob, which can bind and infect cells that express the desmoglein [28]. Third, ONCOS-401 expresses CD40L, which can augment the induction of antitumor responses [31].

Optimization studies of oncolytic adenoviral production processes are essential to enhance and maximize virus productivity and to set conditions for upscaling the purification process, which can provide sufficient yields for clinical studies and the market. Optimization of the manufacturing process of Ad often includes evaluation of the type, confluency, and growth conditions of the host cell line; variation of multiplicity of infection; assessment of optimal harvesting time and initial harvesting method with cell disruption; often treatment with benzonase™ to digest DNA and RNA; and passage through one or more chromatographic columns such as anion exchange, gel infiltration, size exclusion column; and formulation that fosters long-term stability [11].

The purpose of these studies was to optimize the initial steps: conditions for ONCOS-401 production in A549 cells and the initial harvesting processes. Both the growth parameters and the harvesting methods can affect the viral yield. Maximizing the viral production at these initial steps can improve the overall yield after purification. Here, we compared yields for the ONCOS-401 replication and production in T-175 flasks and HYPERFlask flasks, which were used as a scaled-down model for a bioreactor. Although A549 cells do not contain any adenoviral E1 sequences like HEK293 cells, A549 cells support ONCOS-401 replication [32]. Thus, we eliminated the possibility of E1A recombination and generation of E1 wildtype viruses in our stocks by choosing the A549 lung cancer line as the host cell. Modifying the protocols from the T-175 flasks to HYPERFlasks provided a model for scaling up the production. In addition, we compared the viral production by five related harvesting methods. Studies were concentrated on optimization of the multiplicity of infection (MOI), the duration of the incubation period before harvesting, and the initial harvesting method. Comparisons include not only total viral particles from batch but also viral productivity (infectious viral particles/cell and infectious viruses per cm^2^). Optimizing the viral yield in the initial steps provides the necessary foundation for efficient elucidation of the purification methods.

## 2. Results

The two main purposes of these studies were to optimize the harvested viral production for ONCOS-401 in a T-175 and in HYPERFlasks. Cells incubated for 96 h in T-175 or 144 h in HYPERFlask were in a logarithmic phase of growth, almost reaching the maximum of their possible growth. Based on growth curves, cell density at the time for infection (80% confluent) was 220,000 cells/cm^2^.

### 2.1. Effect of Harvesting Time After Infection with MOI of 10

**Figure 1:**
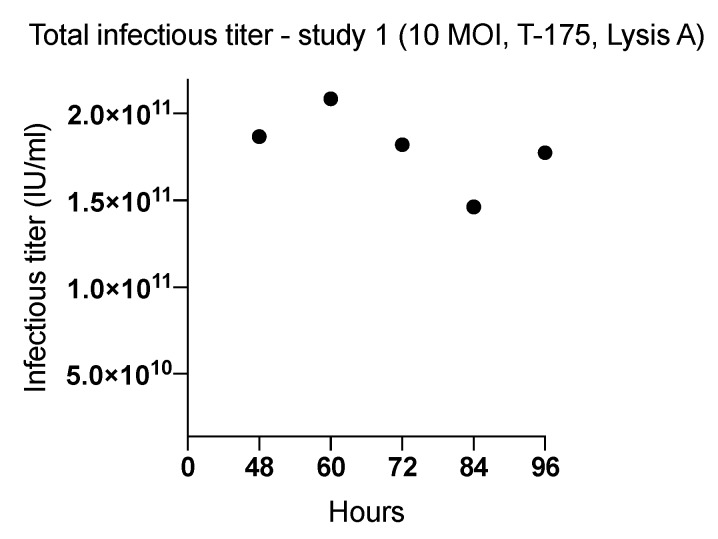
The effect of length of incubation period of ONCOS-401 infected A549 cells (MOI = 10) on the yield of infectious viral particles in T-175 flasks. The infectious titers were tested at harvesting times of 48, 60, 72, 84 and 96 h. The highest viral titers were obtained at 60 h post infection. Values are presented as means.

**Figure 2:**
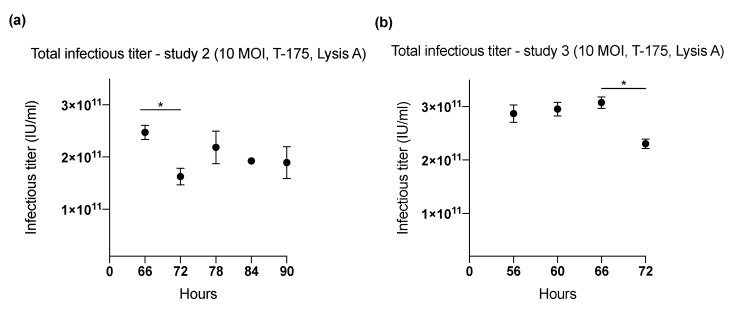
The effect of length of incubation period of ONCOS-401 infected A549 cells (MOI = 10) on the yield of infectious viral particles in T-175 flasks. (**a**) The infectious titers from ONCOS-401 infected A549 cells (MOI = 10) in T-175 flasks were tested at harvesting times of 66, 72, 78, 84, 90 h: the highest viral titers were obtained at 66 h post infection. (**b**) The effect of length of incubation period of ONCOS-401 infected A549 cells (MOI = 10) on the yield of infectious viral particles in T-175 flasks. The yield of infectious viral particles from ONCOS-401 infected A549 cells in T-175 flasks were tested at harvesting times of 56, 60, 66, 72 h: similar titers were obtained at harvest times of 56 to 66 h post infection. Values are presented as means ± SD, * *p* < 0.05.

Three studies (1–3) were performed in T-175 flasks (cell binding surface) to determine the harvesting time (range: 48–96 h) (Figure 1 and Figure 2) that yielded the highest ONCOS-401 viral production (Table 1). The Lysis A harvesting method was performed. The full CPE was observed at 72 h post infection. However, this time point did not result in the highest viral productivity. The three experiments indicated that A549 cells inoculated with a MOI of 10 provided the highest titer at 60 and 66 h (Figure 1 and Figure 2). Thus, the harvest time of 66 h post ONCOS-401 infection was chosen for subsequent experiments.

### 2.2. Effect of Inoculum Size on Quantity of Harvested Viruses

**Figure 3:**
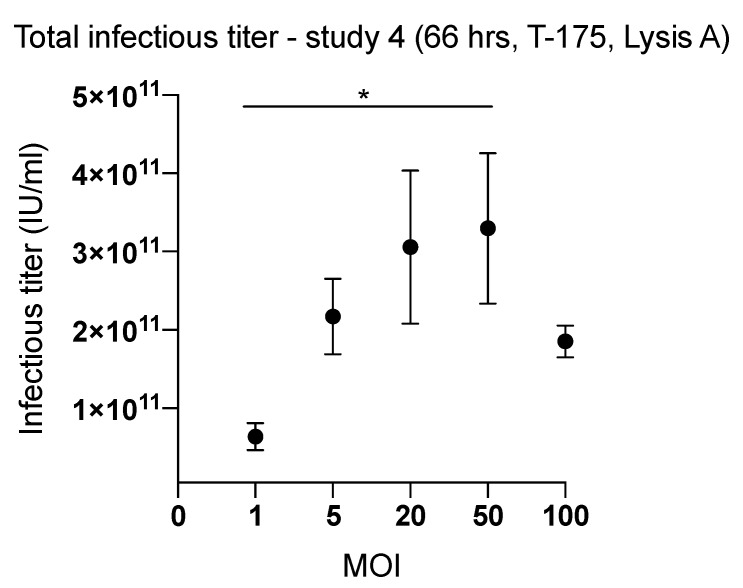
The effect of the input inoculum (MOI) on the yield of infectious viral particles from ONCOS-401 infected A549 cells was determined in T-175 flasks. A549 cells were infected with ONCOS-401 at the indicated MOIs (1, 5, 20, 50 and 100), and the viral particles were harvested at 66 h post infection. Infectious titers were determined by ICC. The A549 cells infected with 20 and 50 MOI provided the highest viral titers. Moreover, one log drop of titer was observed at 1 MOI. Values are presented as means ± SD, * *p* < 0.05.

Comparison of ONCOS-401 yields from T-175 flasks infected with various MOIs indicated a clear dependence of viral productivity on the MOI of the inoculum. Cells infected with 20 MOI or 50 MOI in T-175 flasks followed by the Lysis A harvesting method yielded the highest quantity of harvested virus (Figure 3), 3.06 × 10^11^ and 3.30 × 10^11^ IU respectively. We chose an intermediate MOI, i.e., 30 MOI, for further studies in HYPERFlasks on the production process. We assessed the quantity of harvested ONCOS-401 virus from 3 HYPERFlasks inoculated with a MOI of 30 after 66 h and harvested by the Lysis A method in study 5: the mean viral productivity for the three HYPERFlasks was 14,559 ± 802 viruses per cell (Table 1).

**Figure 4:**
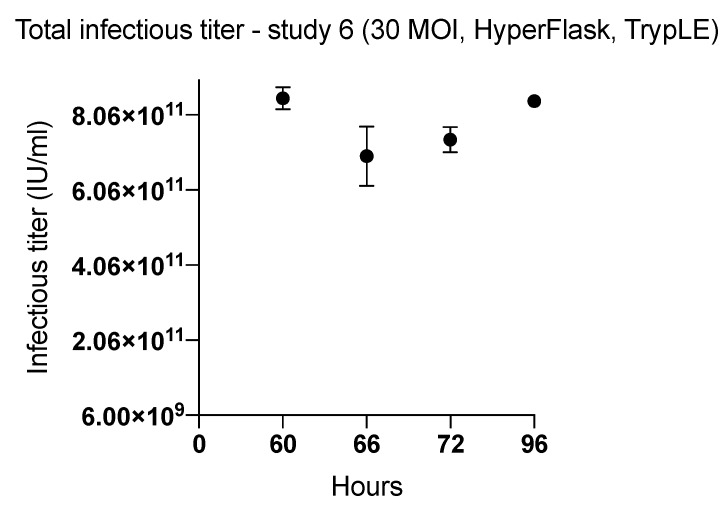
The effect of length of incubation period of ONCOS-401 infected A549 cells (MOI = 30) on the yield of infectious viral particles in HYPERFlasks. ONCOS-401 infected A549 cells (MOI = 30) were incubated in HYPERFlasks for 60, 66, 72, 96 h and the adenoviral particles were harvested by the TrypLE method. The infectious titers were determined by ICC. The yields of infectious ONCOS-401 viral particles were low compared to previous experiments that used the Lysis A method.

Because the inoculum size (MOI = 30) was different from the inoculum in the earlier kinetic experiments, we compared the quantity of harvested virus from cells infected at MOI 30 and incubated in HYPERFlasks at 60, 66, 72, and 96 h incubation time post infection (Figure 4). To theoretically increase the harvest of viral particles from the HYPERFlask, we used the TrypLE harvesting method (Table 2). The viral yields ranged from 2044 ± 189 to 2497 ± 60 infectious viral particles/cell. These yields harvested with the TrypLE method were substantially lower than the viral yields from HYPERFlasks obtained with the Lysis A method in study 5 (14,559 ± 802 infectious viral particles/cell) and 3.02 × 10^12^ ± 0.135 × 10^12^ IU per HYPERFlask.

**Figure 5:**
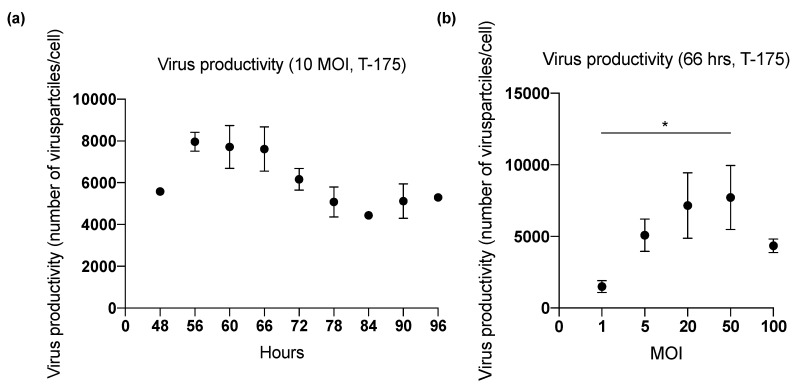
Summary of optimization studies in T-175 flasks. The effect of harvesting time on viral productivity. (**a**) Virus productivity from ONCOS-401 infected A549 cells (MOI = 10) in T-175. (**b**) Virus productivity from ONCOS-401 infected A549 cells in T-175 flasks harvested at 66 h. Values are presented as means ± SD, * *p* < 0.05.

**Figure 6:**
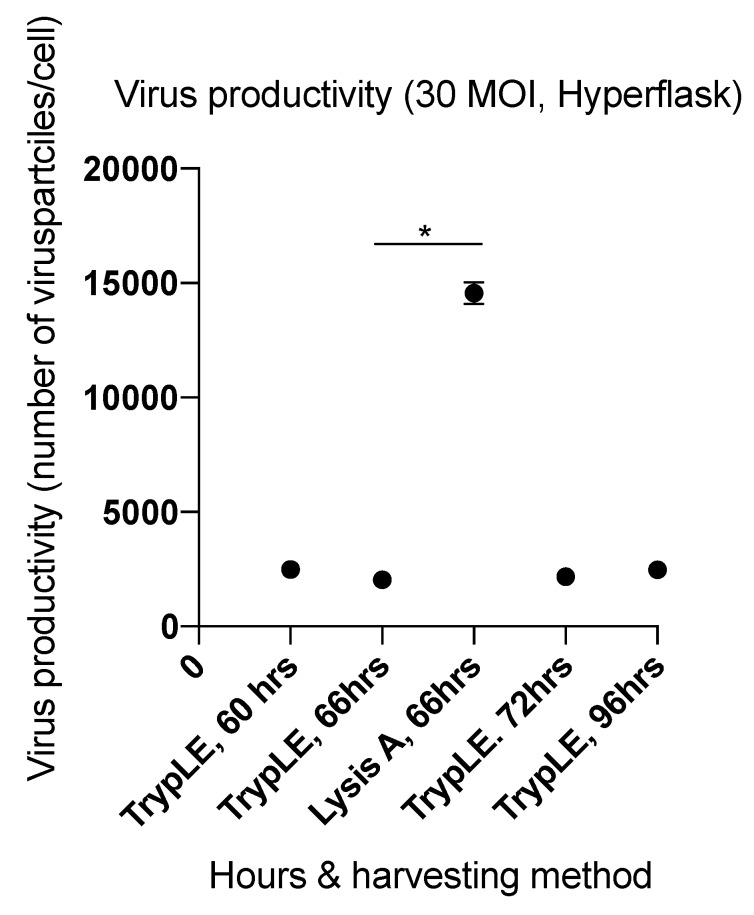
Summary of optimization studies in HYPERFlasks. The effect of harvesting time on total viral infectious titer (IU). Values are presented as means ± SD, * *p* < 0.05.

The pooled optimization studies indicate that the highest viral yields occurred with a MOI of 20 or 50, and an incubation period of 66 h (Figure 5b) for the T-175 flasks, and with a MOI of 30 and an incubation period of 66 h in the HYPERFlask harvested with Lysis A (Figure 6).

### 2.3. Factors Affecting Yield in the Harvesting Method

Viral productivity is dependent not only on efficient virus infection and replication but also on the method for harvesting the infected cells and releasing the viruses (Table 2). Since physical disassociation is more challenging in HYPERFlasks, efficient lysis buffers are essential for maximizing viral yield. Lysis buffer allowed collection of a mean harvest for HYPERFlasks in study 5 (MOI of 30; harvesting time 66 h) of 14,559 ± 802 viral particles per cell. Assuming that TrypLE buffer would maintain or augment the harvest, study 6 both investigated the indicated harvesting times and modified the harvesting method to include TrypLE. However, the harvested viral production in study 6 ranged from 2044 ± 189 to 2497 ± 60 viral particles/cells and was the lowest among all performed studies. We hypothesized that many viral particles remained in the pellet during the TrypLE- based harvesting process.

To determine the role of the harvesting method in the low yield, we used three different harvesting methods on T-175 flasks in study 7. The viral yields of the T-175 flasks harvested by the mechanical and Lysis buffer A method (10,887 ± 100 viral particles per cell) were approximately 100% higher than the viral yield from the Lysis B method (no TrypLE; 4331 viruses/cell) or the Lysis C method (no TrypLE, with washes and benzonase; 5741 viral particles per cell). Comparison of the viral yields from the T-175 flasks harvested by the Lysis B and Lysis C methods raised the possibility that additional washes of the T-175 flask combined with benzonase treatment (Lysis C) augmented the viral yield up to 32% compared to Lysis B method. The TrypLE and Lysis D methods were not assessed in T-175 flasks. The harvesting methods of ONCOS-infected A549 cells grown in T-175 flasks ranked for viral yields were Lysis A >> Lysis C > Lysis B.

The viral yields from HYPERFlasks harvested with Lysis C (washes, benzonase) and the Lysis D (washes) method resulted in an approximately 45% lower viral yield (1275 viruses/cell) than the viral yield from HYPERFlask harvested by Lysis buffer C-wash-benzonase method (2374 viruses/cell). Since we hypothesized that many viral particles remained in the pellet during the TrypLE-based harvesting process, we assessed the cell debris pellet from the second 4000 rpm centrifugation for unreleased viral particles during Lysis D harvesting method of HYPERFlask. The pellet was resuspended in 10 mL of Lysis buffer supplemented with benzonase and incubated at 37 °C for 1 h (Lysis buffer D). After the lysate was vortexed to ensure a homogenous suspension, it was clarified by 4000 rpm centrifugation. The viral yield released from the pellet was 1275 viruses/cell (pellet 1). These results confirmed that the 4000 rpm pellet had retained about half of the viral productivity from the Lysis D harvesting method. To determine if most of the viral particles had been released during the first treatment of pellet 2, these steps were repeated on the final pellet 2. The viral yield from Pellet 2 was 315 viruses/cell, which indicated that 80% of the viruses in pellet 1 had been released into the supernatant by the treatment. Taken together, the harvesting methods used for the HYPERFlasks were ranked by viral productivity as follows: Lysis A > Lysis C > TrypLE > Lysis D (Table 1 and Table 2).

### 2.4. Viral Productivity Summary

The viral production conditions in both T-175 flasks and HYPERFlasks included A549 cells that were seeded at 15,000 cells/cm^2^ and grown to 220,000 cells/cm^2^ (approximately 80% confluent), ONCOS-401 infection at 30 MOI, and an incubation period of 66 h. The Lysis A harvesting method provided the highest viral yield from both T-175 flasks (10,887 ± 100 infectious viral particles/cell) and HYPERFlasks (mean 14,559 ± 802 infectious viral particles/cell).

### 2.5. Glucose and Lactate Measurements

Glucose and lactate measurements showed stable and comparable conditions for cell growth among flasks represented in each study. The lactate measurements ranged from 88.0% to 106.8% of control. The mean glucose measurements ranged from 94.4% to 106% of control values. These values indicated that the A549 cell cultures, regardless of infection, were not limited by low glucose levels nor by excess lactate levels.

## 3. Discussion

Common challenges for manufacturing clinical grade oncolytic adenoviruses include the optimization of growth conditions (e.g., cell line, media, and vessel), viral productivity, and initial yields; scalable purification strategies; and formulations that provide long-term stability [11]. Safety considerations during production of clinical viral seed stocks or clinical viral product require a host cell line that minimizes or eliminates the possibility of recombination with host cell integrated Ad E1A sequences that can generate wildtype E1A-driven adenoviruses. The recombined wildtype E1A-driven adenoviruses often have a growth advantage during production of clinical grade viruses and expand to a higher percentage of the viral yield with each passage. As contaminating wildtype E1A-driven viruses can replicate in healthy cells rather than being restricted to growth in cancer cells, the contaminating wildtype E1A-driven viruses may change the specificity of the administered viral product in vivo. On review, HEK293 cells are often used for adenovirus research and production of replication-defective adenoviral vectors as they contain and express the integrated *E1A* genes [33]. However, ONCOS-401 does not need exogenous E1A to grow in human tumor cells [32]. Thus, the use of HEK293 cells during production of clinical grade ONCOS-401 carries the potential unnecessary risk of generation of wild type E1A-driven adenovirus contamination [11]. To avoid this safety issue, we chose to use the A549 cell line, which is suitable for adenovirus production of replicating adenovirus vectors [34] (like ONCOS-401) that do not require complementation of the *E1A* genes from the host cell.

The conditions that led to the highest ONCOS-401 production in this series were A549 cells grown to 220,000 cells/cm^2^, ONCOS-401 infection at 30 MOI, and an incubation period of 66 h. The Lysis A harvesting method provided the highest viral yield from both T-175 (175 cm^2^, 10,887 ± 100 infectious viral particles/cell) and HYPERFlasks (1720 cm^2^; mean 14,559 ± 802 infectious viral particles/cell). These results are substantially higher than the yields in two bioreactors that had grown A549 cells on microcarrier beads and infected with Onco-Ad5 at MOI = 10 [34]. After 72 h, the two bioreactors had generated 630 ± 310 infectious particles (ip)/ cell and 260 ± 50 ip/cell, which Souza et al. [34] reported as similar to previous reports [35,36]. The viral productivity of these two bioreactors [34] was routinely 4 to 10 times lower and up to 20- to 50-fold lower than the results reported here. However, the different assays used to measure infectious viral particles (TCID50 [34] and ICC [20]) may contribute to the different reported viral productivities.

Lesch et al. [37] reported that after HEK293 cells reached 70–80% confluency, they infected with wildtype Ad5 as a model for optimization of viral productivity from bioreactors. The A549 cell density in these studies were infected at similar confluency (approx. 80%) but the actual number of A549 cells was higher (220,000 cells/cm^2^) than the number of HEK293 reported by Lesch et al. (173,000 HEK293 cells/cm^2^). Lesch et al. reported [37] that the total yield of wildtype Ad5 (MOI 200) in HEK293 cells harvested by Lysis buffer was 7.3 × 10^13^ viral particles in an iCell bioreactor with a surface area of 7362 cm^2^ or 9.9 × 109 viral particles/cm^2^. Their different harvesting methods yielded viral productivity that ranged from 1.6 × 10^9^ vp/cm^2^ to 9.9 × 10^9^ viral particles/cm^2^. In comparison, the ONCOS-401 infected A549 cells grown in HYPERFlasks and T-175 flasks here had produced up to 1.75 × 10^9^ ± 0.08 and 2.12 × 10^9^ ± 0.2 infectious particles of ONCOS-401 per cm^2^ of surface area, respectively. Although the detection of anti-hexon-stained cells by DAB in our ICC method [20] may be less sensitive than detection by FACS [37], wildtype Ad probably also shows greater replicative ability in HEK293 cells, which express more E1A than ONCOS-401 shows in A549.

The harvesting methods also can have a major impact on viral yield. The Lysis A harvesting method utilized benzonase, which is widely used to cleave DNA and RNA and support release of viruses [35,38]. Viral productivity of ONCOS-401 infected A549 cells (MOI = 10, 66 h incubation) grown in T-175 flasks and harvested with Lysis A buffer method was 6682 ± 300 and 8538 ± 248 infectious viral particles/cell. A higher inoculum of MOI 30 increased viral productivity by 28% to 63%, providing 10,887 ± 100 infectious viral particles/cell. To further investigate harvesting methods, ONCOS-401 infected A549 cells (MOI = 10, 66 h incubation) were grown in T-175 flasks and harvested by the Lysis B or C methods. The viral productivity from flasks harvested by Lysis B or C were 4331 and 5741 infectious viral particles/cell, respectively. In comparison, use of the Lysis A harvesting method provided viral productivity of 10,887 ± 100 infectious viral particles/cell which is 152% and 89% greater than the yield with the Lysis B and Lysis C methods, respectively. Similar to the T-175 viral yields, the ONCOS-401-infected A549 cells (MOI 30) grown in HYPERFlasks (66 h incubation) and harvested by the Lysis A buffer method yielded the highest viral productivity: mean 14,559 ± 802 infectious viral particles/cell. In comparison, the TrypLE harvesting method of ONCOS-401-infected A549 cells (MOI 30) grown in HYPERFlasks for 66 h yielded a much lower viral productivity: 2044 ± 189 infectious viral particles/cell. Taken together, our results support the continued usage of the Lysis A harvesting method with benzonase, in agreement with [11,30,35,38]. However, efficient methods for viral purification that removes benzonase may be necessary.

The limitations of this study include four points to consider. First, these optimal growth conditions for A549 may need to be modified for other cell lines and other recombinant Ad that do not utilize the same Ad5/3 fiber knob and thus do not share receptors. Kawakami et al. showed that incorporation of the Ad5/3 fiber knob increased viral replication compared with its Ad5 construct [39]. Second, these optimal MOI and harvesting times may vary for other oncolytic adenoviruses, even if they carry the chimeric Ad5/3 fiber knob. For example, the expression of a different transgene by the oncolytic adenovirus, a different mutation of E1A, or a different promoter driving expression of the mutated E1A protein may modify the kinetics of viral replication [40]. In contrast, Doloff et al. showed that Ad5-hTERT-E1A driven oncolytic adenovirus replicated less efficiently than its Ad5 counterpart, ONYX-015 [40]. Diaconu previously reported the initial characterization of ONCOS-401 virus, comparing its cell cytotoxicity to the Ad5/3-hTERT-E1A virus, i.e., without the hCD40L in the E3 region [23]. They showed that the Ad5/3hTERT-E1A virus had much greater cell cytotoxicity activity at 48 h than ONCOS-401 [32], which is consistent with the later harvesting times of ONCOS-401 observed here. Third, oncolytic viruses may vary in their lytic ability against the host cell line and may necessitate including viral particles from the initial harvest supernatant, as done in the Lysis A harvesting method but not in the TrypLE, Lysis B, C, and D harvesting methods described here. Ease of downstream purification steps may be dependent on the volumes obtained during these initial steps; some harvesting methods allow adjustment of the volume for resuspension of harvested infected cells such as the TrypLE, Lysis B, Lysis C-wash, Lysis buffer D-benzonase methods described here. In addition, initial concentration of the oncolytic adenovirus from large initial volumes that include washes may be enhanced by binding to and elution from a column containing an engineered dual-receptor protein [41]. Fourth, although additional adjustments may be needed to optimize yields in vertical wheel bioreactors [34], optimization of many of the presented parameters, as well as key hydrodynamic parameters, can aid in elucidation of the most effective conditions for non-planar bioreactors.

Despite these limitations, this stepwise process of identification of optimal conditions of cell growth, viral replication, and initial processing may be applicable to the optimization of the manufacturing of other oncolytic adenoviruses, whether they are used as single agents [9,11,42,43,44] or in combination therapies [3,6,20,45,46].

## 4. Materials and Methods

Studies were focused on optimization of the following parameters: cell density (determined previously), multiplicity of infection (MOI) of inoculum of ONCOS-401 and harvesting time (time post infection). An additional aim was to adjust conditions and technical aspects of virus harvesting to maximize the productivity in HYPERFlasks.

### 4.1. Cells

A549 cells were grown in Optipro SFM (Gibco, #12309-019) supplemented with 1% fetal bovine serum (FBS, Gibco, 10099-141) and 4 mM Glutamax (Gibco, #35050-038) at 37 °C in 5% CO_2_ in humidified air. Cells were passaged after trypsin treatment with TrypLE (Gibco, #12563-011). Cells were used at passage 94 or later (WBC Vibalogics) for the described experiments. A549 cells were seeded at 15,000 cells/cm^2^ in Corning CellBind T-175 flasks (#3292) or HYPERFlask (Corning, #10030) and incubated for 96 h or 144 h, respectively. Subsequent incubations of cells or viruses were performed at 37 °C in 5% CO_2_ in humidified air unless indicated otherwise.

### 4.2. Lactate/Glucose Measurements

Supernatant samples were collected before infection and at time of harvesting to evaluate growth conditions in flasks. The lactate and glucose concentrations were measured by using the Accutrend and its meter (OT-134), according to the manufacturer’s instructions (Roche, Cobas^®^).

### 4.3. Virus Titration

Quantitation of infectious viral particles were performed by the previously described immunocytochemistry (ICC) assay [20], (Figure S1). Briefly, infected cells were detected at 24 h post infection by staining with anti-hexon antibody and detection by DAB. These reagents—anti-Ad hexon, Novus Biologicals, NB600-413; Biotin-SP-conjugated goat anti-mouse IgG, Jackson Immuno-Research 115-065-062—were used. Viral productivity was defined as the total number of harvested viruses (infectious titer) divided by the total number of cells.

### 4.4. Cell Counting

One flask was harvested at the time of infection for quantifying cell counts and determining lactose and glucose concentrations. After collecting the supernatant, the T-175 flask and HYPERFlask were rinsed gently with 50 mL or 100 mL of OptiPro (without FBS), respectively. The T-175 flask and HYPERFlask cells were treated with 5 mL TrypLE or 50 mL of TrypLE, respectively, for approximately 1–5 min. The efficiency of TrypLE detachment in the HYPERFlasks was assessed visually under a microscope (Evos xl, OT-114). If needed, HYPERFlasks were treated again with TrypLE.

### 4.5. Viral Infection

The cells were counted by a cell counter (Countess, OT-115), the quantity of ONCOS-401 was calculated for infection of the T-175 or HYPERFlasks. The flasks were incubated 4 h, flasks received 46 mL of media or 460 mL media with 1% FBS and were incubated for the indicated time periods.

### 4.6. Harvesting Methods

The kinetic studies (48–96 h post infection with ONCOS-401) were performed in order to find the most optimal time for virus harvesting and maximizing the viral yield. Trypsinizing was implemented to release any infected cells that remained attached to the vessel at the time of harvesting and further enhance the viral yield.

Two basic methods of harvest were used: Lysis buffer A and TrypLE. Three subsequent methods combined steps from both the Lysis A method and the TrypLE method: Lysis B, Lysis C-wash, and Lysis buffer D-benzonase methods (Table 2).

Studies 1–5 used the Lysis A harvesting method: media samples were collected. Warm (37 °C) chemical lysis buffer supplemented with benzonase (15 U/mL, sigma-Aldrich, E1014-5KU) was added to each T-175 flask (10 mL) and HYPERFlask (100 mL). After 1 h incubation, cells were collected in sterile Falcon tubes and centrifuged at 3000 RPM for 10 min at + 4 °C. Supernatants were collected and sucrose was added to 5% into the virus suspension. Viral stocks were stored at −80 °C.

TrypLE harvesting method: The medium samples and cells were collected. To remove remaining attached cells from the growing surface of the HYPERFlask (viewed via microscope), we added 50 mL of TrypLE to each HYPERFlask and incubated them for 5 min. HYPERFlasks were tapped firmly 5–10 times to increase cell detachment. Residual cells were washed and collected with 50 mL of OptiPro media. If indicated, adherent cells before the addition of TrypLE were washed with 100 mL of OptiPro (without PBS). After cells were detached, the contents of the HYPERFlask were collected (50 mL-Falcon tubes) and centrifuged gently at 1000 RPM for 10 min at 4 °C (centrifugation 1). After the supernatant was gently removed, the fragile pellet was resuspended into 8–10 mL of OptiPro without FBS. This solution was used to resuspend the pellets from all centrifuged samples from a given HYPERFlask. The concentrated viral sample was pipetted back and forth until suspension became homogeneous. The samples were frozen in 10 mL aliquots (15 mL sterile Falcon tubes) at −80 °C. Samples were thawed and frozen three times: room temperature water bath (about 15 min), vortexed, and returned to −80 °C freezer for at least overnight. After the third thawing and vortexing, the lysate was centrifuged at 4000 rpm for 15 min at + 4 °C (pellet 2). The supernatant was collected, and sucrose was added to yield a 5% solution. Aliquots were stored at −80 °C.

Lysis B harvesting method followed the TrypLE method, with the following two modifications: no TrypLE was added, and no subsequent hour incubation was performed.

The Lysis C-wash harvesting method followed the Lysis B harvesting method with one wash for T-175 (50 mL OptiPro with no FBS) and two washes of HYPERFlask (100 mL) as part of viral and cell collection and before centrifugation.

Lysis D–benzonase harvesting method followed the Lysis C harvesting method with three modifications. Cells were washed with media with no FBS; HYPERFlask, 2 × 100 mL. The pellet collected after first centrifugation was resuspended with 10 mL lysis buffer supplemented with benzonase (15 U/mL) and was incubated for 1 h at 37 °C. Subsequently, viral suspension was mixed by vortexing to ensure a homogeneous suspension.

### 4.7. Biostatistics

The mean number of infected cells per field was determined from evaluation of 30 photographs. The 95% confidence interval (CI) for ICC was calculated based on the numbers of infected cells per field by using T-Distribution analyses (Student’s T Distribution), where SL was the significance level of 0.05, SIZE was the number of fields of the group (30 photos represents one field) and SD was the standard deviation for each field (30 photos). Precision was defined as the percent coefficient of variation (%CV). We calculated %CV by dividing the standard deviation by the mean for a number of replicate determinations. %CV was calculated based on the numbers of infected cells per field (30 photos/field). Statistical analysis was performed with GraphPad Prism software version 8 (La Jolla, CA, USA). One-way ANOVA (Kruskal-Wallis test & Dunn’s multiple comparisons test) was used to compare groups.

## Figures and Tables

**Table 1:** Virus Productivity.

Optimization Study (Number)	Harvesting Time (h)	MOI	Harvesting Method	Total Virus Particles (IU), (Mean)	Virus Productivity (Number of Virus Particles/Cell), (Mean)
1 (T-175)	48	10	Lysis A	1.86 × 10^11^	5576
	60			2.08 × 10^11^	6226
	72			1.82 × 10^11^	5438
	84			1.46 × 10^11^	4365
	96			1.77 × 10^11^	5299
2 (T-175)	66	10	Lysis A	2.47 × 10^11^	6682
	72			1.63 × 10^11^	3779
	78			2.19 × 10^11^	5083
	84			1.92 × 10^11^	4476
	90			1.90 × 10^11^	5124
3 (T-175)	56	10	Lysis A	2.87 × 10^11^	7966
	60			2.95 × 10^11^	8205
	66			3.07 × 10^11^	8538
	72			2.30 × 10^11^	6401
4 (T-175)	66	1	Lysis A	6.39 × 10^10^	1494
		5		2.17 × 10^11^	5085
		20		3.06 × 10^11^	7153
		50		3.30 × 10^11^	7715
		100		1.85 × 10^11^	4338
5 (HYPERFlask)	66	30	Lysis A	3.02 × 10^12^	14,559
6 (HYPERFlask)	60		TrypLE	8.51 × 10^11^	2497
	66			6.96 × 10^11^	2044
	72			7.40 × 10^11^	2172
	96			8.43 × 10^11^	2475
7 (T-175 & HYPERFlask)	66	30	Lysis A T-175	3.72 × 10^11^	10,887
			Lysis B T-175	1.48 × 10^11^	4331
			Lysis C T-175	1.96 × 10^11^	5741
			Lysis D HYPERFlask	4.36 × 10^11^	1279
			Lysis C HYPERFlask	8.09 × 10^11^	2374
			Benzonase treatment (pellet 1) HYPERFlask	4.35 × 10^11^	1275
			Benzonase treatment (pellet 2) HYPERFlask	1.08 × 10^11^	315

**Table 2:** Comparison of the Harvesting Methods.

Steps	Lysis A	TrypLE	Lysis B	Lysis C	Lysis D
**Collected supernatant**	Y	Y	Y	Y	Y
**Chemical lysis buffer with benzonase treatment (1 h)**	Y	N	N	N	N
**Washes (1–2 x)**	N	Y	Y	Y	Y
**TrypLE**	N	Y	N	N	N
**Mechanical disassociation (firmly tapped 5–10 x)**	N	Y	Y	Y	Y
**TrypLE**	N	Y	N	N	N
**Centrifugation**	N	1000 rpm	1000 rpm	1000 rpm	1000 rpm
**Supernatant removed & discarded**	N	Y	Y	Y	Y
**Pellet saved & resuspended in 8–10 mL**	N	Y	Y	Y	Y
**Chemical lysis buffer with benzonase** **(1 h) on pellet**	N	N	Y	Y	N
**Pipette-based disruption and homogenization**	N	Y	Y	Vortexed	Vortexed
**Frozen −80 °C, thawed 3 x**	N	Y	Y	Y	Y
**Centrifugation**	3000 rpm	4000 rpm	4000 rpm	4000 rpm	4000 rpm
**Supernatant retained, 5% sucrose, & frozen at −80 °C**	Y	Y	Y	Y	Y
**Viral titer by ICC**	Y	Y	Y	Y	Y

## Supplementary Materials

Supplementary materials can be found at http://www.mdpi.com/1422-0067/20/3/621/s1. Figure S1 ICC-staining (10x objective).

## Abbreviations

DAMP	damage-associated molecular patterns
DSG	desmoglein
GM-CSF	granulocyte-macrophage colony-stimulating factor
hTERT	human telomerase promoter
ICC	immunocytochemistry assay
i.v.	intravenously
MOI	multiplicity of infection
OV	oncolytic viruses
