# Supplementary material for: Optimization of Early Steps in Oncolytic Adenovirus ONCOS-401 Production in T-175 and HYPERFlasks
Source: Int J Mol Sci. 2019 Jan 31;20(3):621. doi: 10.3390/ijms20030621 (PMC6387112; doi:10.3390/ijms20030621)
Supplement: Supplementary file 1 [file ijms-20-00621-s001.pdf]

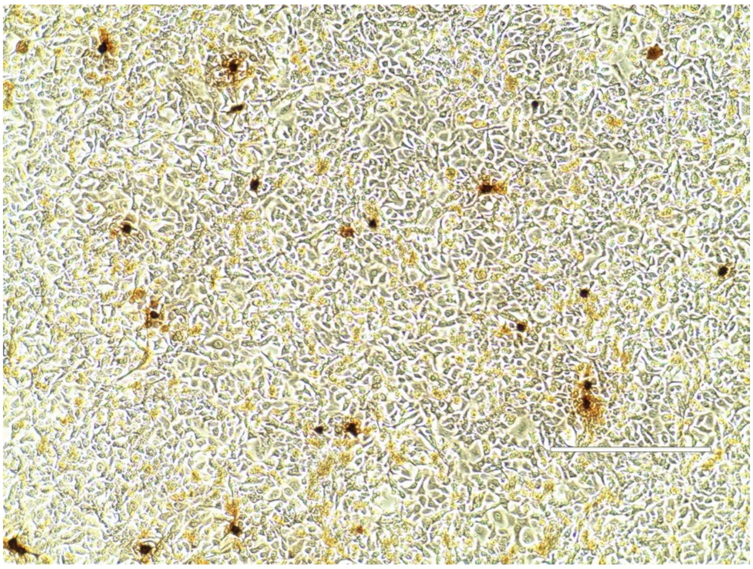

Figure S1. ICC staining (10x objective). An example of the ICC staining (72 hours post infection with ONCOS-401). Dark spot indicates A549 cell infected with ONCOS-401. The determination of the infectious titer is based on visual quantification of infected A549 cells. The target molecule in the assay is the virus hexon protein, an antigen associated with the ONCOS-401. Upon the infection, the cell synthesizes excessive amounts of hexon proteins, which are targeted by anti-hexon antibody. This mouse-origin antibody, in turn, is targeted by the 2nd Biotin-SP-conjugated antibody, which functions as a platform for Extravidin-peroxidase. Upon the addition of the stain, DAB is oxidized by peroxidase, forming an insoluble brown precipitate, which gives the infected cells an intense dark color.
